# Developing a perinatal palliative care service package for women with fetal anomaly diagnosis: protocol for mixed methods study

**DOI:** 10.1186/s12978-020-0881-8

**Published:** 2020-03-04

**Authors:** Ziba Raisi Dehkordi, Shahnaz Kohan, Maryam Rassouli, Elahe Zarean, Azadeh malekian

**Affiliations:** 1 Faculty of Nursing and Midwifery, Isfahan University of Medical Sciences, Isfahan, Iran; 2 Nursing and Midwifery Care Research Center, Faculty of Nursing and Midwifery, Isfahan University of Medical Sciences, Isfahan, Iran; 3 School of Nursing & Midwifery, Shahid Beheshti University of Medical Sciences, Tehran, Iran; 4 Department of Obstetrics and Gynecology, Fetal Medicine Unit, Isfahan University of Medical Sciences, Isfahan, Iran; 5 Psychosomatic Research Center, Isfahan University of Medical Sciences, Isfahan, Iran

**Keywords:** Perinatal palliative care, Service package, Fetal anomaly, RAND method, Mixed method, Protocol for study

## Abstract

**Background:**

Diagnosis of perinatal anomalies is a stressful experience that can negatively affect mothers, families, health-care systems, and societies. Perinatal palliative care (PPC) is a new development in maternity services which focuses on emotional, spiritual, social, and symptom management and provides care for women and families with fetal anomaly diagnosis. Therefore, this study aimed to develop a service package for women with fetal anomaly diagnosis in socio-cultural context of Iran.

**Methods:**

This research is an exploratory mixed methods study with the qualitative-quantitative sequencing design that consists of four sequential phases. In the first phase, following a qualitative approach, the researcher will explore the needs and experiences of women with fetal anomaly diagnosis, their families, health care providers and policy-makers. At the second phase, based on the review of the literature, Program, guideline, service package and protocol for care of women and their families after perinatal anomaly diagnosis will be identified in other countries. In the third phase, recommendations from qualitative phase and literature review will be combined, the initial protocol of the palliative care service package for perinatal anomaly diagnosis will be identified and prioritized. In the fourth phase, the opinion of experts about this service package will be collected by using RAND/UCLA Appropriateness Method technique and the applicability of the service package’s recommendations in clinical settings will be determined.

**Discussion:**

The results of this Mixed Methods study are expected response the needs and experiences of the women with perinatal anomaly diagnosis being met in the socio-cultural context of Iran and a service package for palliative care of these women developed.

## Plain English summary

Pregnancy with fetal anomaly diagnosis is a stressful experience with impacts on individuals, families, health-care systems, and societies. Considering the lack of a perinatal palliative care service package for the women with Perinatal anomaly diagnosis in Iran and also the challenges that these mothers face, the findings of this study will provide suitable sources of information for the required interventions to wellbeing and palliative care of these women. This study is an exploratory study with mixed method design (qualitative-quantitative) that consists of four consecutive phases. Following a qualitative approach, the researcher will explore the needs and experiences of the women with fetal anomaly diagnosis, their families, health care providers and policy-makers. In the second phase, based on the review of the literature, Program, guideline and protocol for care of women and their families after perinatal anomaly diagnosis will be identified in other countries. In the third phase, recommendations from qualitative phase and literature review will be combined, the initial protocol of the palliative care service package for perinatal anomaly diagnosis will be identified and prioritized. In the fourth phase, the opinion of experts about this service package will be collected by using RAND/UCLA Appropriateness Method technique and the applicability of the service package’s recommendations in clinical settings will be determined. Ultimately, a perinatal palliative care service package will be provided.

## Background

Recent technological advances in prenatal screening led to an increasing number of fetal anomalies to be diagnosed [1]. The use of ultrasound imaging and maternal serum marker screening to detect aneuploidies and other birth defects are a routine part of prenatal care in the first and/or second trimesters in many countries [2]. Fetal anomalies are defined as structural or functional anomalies (e.g. metabolic disorders) that occur during intrauterine life and can be identified prenatally, at birth, or sometimes may only be detected later in infancy [3]. European Surveillance of Congenital Anomalies (UROCAT) recorded a total prevalence of major congenital anomalies of 23.9 per 1000 births [4]. In Iran, the prevalence of fetal anomaly has been estimated to be 18/1000 live births [5]. Owing to the increased use of prenatal screening for fetal anomalies and the legal restrictions on abortion in such cases, women are confronted with making crucial decisions for their fetus [6]. Fetal anomalies create a challenge for families to choose between continuation of pregnancy or elective termination of it [7] .Factors contributing to the complexity of this decision-making are an array of psychological, social, ethical/moral and the family personality, family background, and religious beliefs [8]. When the pregnancy is terminated, women show post-traumatic stress, prolonged grief, depression, anxiety, concern about future, and sense of failure [9–11]. Decision for terminating pregnancy in the second trimester is very difficult and complicated, also the limitations in the diagnosis of fetal anomalies in this trimester increase the complexity of this decision-making [12]. Parents deciding to continue pregnancy following diagnosis of fetal abnormality experience grief, high levels of anxiety and depression during pregnancy, worries about the status of continuing pregnancy, dangers of planned surgical treatment, status of the baby after birth, and the care of other children during the period after birth [11, 13–16]. They need access to the information or services women’s for decreasing fear, stress and anxiety and facilitate coping with the fetal anomaly diagnosis as they continue their pregnancies and are prepared for the birth of their children [11, 17]. Studies showed when perinatal palliative care services are available during this critical decision-making time period, parents may choose to continue the pregnancy [18–20]. Perinatal palliative care is a new development in maternity services that focuses on physical, emotional, psychosocial, social, and spiritual needs of parental and families during the pregnancy, childbirth and postpartum period when a fetus or a newborn has an identified anomaly [19, 21]. Perinatal palliative services with a multi-professional and interdisciplinary team approach is beginning from anomaly diagnosis to the time of traditional bereavement care [21–23]. Providing perinatal palliative care comprehensively improve the quality of life of families, thus integrating this care into the routine maternal and newborn services can lead to efficient utilization of healthcare resources. Therefore, it is important for palliative care policy makers to explore the needs of women, family, and staff, how to best deliver effective care, and how to design and implement effective public policies [24].

As there is not a perinatal palliative care service package for these women in Iran’s health system, and also considering the challenges that these women face, this research aim is to develop a Perinatal Palliative Care Service Package for women who continue pregnancy after a fetal anomaly diagnosis, during pregnancy, delivery, and the postnatal period.

### Objectives

The objectives of each phase are as follows:

### The first phase: qualitative study

Exploring the needs and experiences of women with fetal anomaly diagnosis, their families, health care providers and policy-makers.

### The second phase: review of the literature

1. Determining perinatal palliative care service package, Program, guideline and protocol in the other countries
2. Extracting perinatal palliative care strategies and recommendation from literature

### The third phase: designing a preliminary perinatal palliative care service package

1. Integrating the results of the qualitative phase and literature review
2. Designing the initial palliative care service package for women with fetal anomaly diagnosis

### The fourth phase

1. Determining the opinion of experts about palliative care service package by using RAND/UCLA Appropriateness Method technique
2. Determining applicability of the service package in clinical settings

## Methods/design

This is a sequential exploratory Mixed Methods study approved by the Isfahan University of Medical Sciences Ethics Committee (IR.MUI.REC.1398.357).

This study consists of four sequential phases. Following a qualitative approach, the researcher will explore the needs and experiences of women with fetal anomaly diagnosis, their families, health care providers and policymakers. Data will be collected through deep, semi-structured interviews and taking field notes. The participants who are eligible for participating in the study will be selected using purposive sampling method and with maximum variation. Simultaneously, while collecting the data, the interviews will be analyzed using a conventional qualitative content analysis method. Sampling will be continued until data saturation occurs.

In the second phase, based on the review of the literature, Program, guideline, service package and protocol for caring for women and their families after perinatal anomaly diagnosis will be identified in other countries. In the third phase, combining the results of the qualitative phase and literature, the initial recommendation of palliative care service package for perinatal anomaly diagnosis will be identified and prioritized. Then, a preliminary service package will be designed based on these data. In the fourth phase, the opinion of experts about this service package will be collected by using RAND/UCLA Appropriateness Method technique [25] . Then applicability of the service package recommendations in clinical settings will be determined.

### Phase I: qualitative study

The first phase of this study will explore the needs and experiences of women with fetal anomaly diagnosis and their families, health care providers and policy-makers. In this research, qualitative study will be carried out using conventional content analysis method [26].

#### Sampling method

The participants will be selected by purposeful sampling method and with the maximum variation regarding the age, education, social status, number of pregnancies and deliveries, job, gestational age**,** and the time of diagnosis and decision making; continuing the pregnancy in the prenatal period; child birth; during the newborn’s life and death, during the early postpartum and postpartum period.

The participants:

In the qualitative phase of the present study, the research population will consist of the women and their families with diagnosis of fetal anomaly during the prenatal period who have referred to hospitals, health centers and clinics, health care providers including obstetricians, geneticists, neonatologist, midwives, nurses, general practitioners, sonographists, perinatologist reproductive health professionals, and maternal health policy makers.

Inclusion criteria for participants:

a) Women with fetal anomaly diagnosis during perinatal period will be included in the study by the following criteria:

1. Having informed consent to participate in the research.

2. Being able to communicate and conduct interviews.

3. Iranian citizenship with the ability of understanding and speaking Persian.

4. Continue their pregnancy either voluntarily or imposed by the law.

b) Health providers and maternal health policy makers’ criteria.

1. Participate in the study with informed consent.

2. Those who have had at least one experience of providing health and medical services for mothers with fetal anomaly diagnosis.

#### The research setting

Pregnant women with fetal anomaly diagnosis who come for a visit (prenatal clinic and health center) or their newborn in neonatal intensive care unit (NICU) or maternity care unit will be selected purposefully.

Interviews will be conducted at the time and place in agreement with the participants at hospital, health centers, midwifery or obstetricians` office, pediatrics’ office, prenatal clinics, work places, university, home, etc.

#### Data collection in the first phase (qualitative)

In this phase, data will be collected through in-depth and semi-structured interviews and field noting will continue until data saturation. Using purposive sampling method and maximum variation, the eligible participants will be invited to join the research. After introducing the objects and methodology of the research and obtaining written informed consent from the participants, the interviews will be conducted in a private and comfortable environment, place and time of the interview will be selected in agreement with the participants. Interviews will be conducted by guiding questions. The interviews will be recorded and transcribed as soon as possible.

#### Data analysis of the first phase (qualitative)

Conventional content analysis approach will be used for data analysis [26]. After transcribing each interview, the text will be read line by line, and its meaning units will be identified. Then, the sentences and the important phrases will be underlined, and the main ideas derived from them are labeled as codes. After extracting the initial codes, data reduction will be done, and eventually subcategories, categories and the main categories will respectively appear from these codes. A computer-assisted program MAXQDA 18 is used to manage the data [27].

#### Rigor and trustworthiness of the qualitative data

In order to assure the rigor of the data, four criteria are suggested: credibility, dependability, transferability and confirmability [28].

To ensure the credibility of the study, the participants will be selected with the maximum variation. Spending a sufficient time for data collection, and the integration of multiple data collection methods, such as interview and taking notes in the field will be used. The participants will review the extracted codes. To confirm the reliability of the findings, some examples of code extraction and narratives will be reviewed by an external observer in order to control the accuracy of the researcher’s perception. In order to increase the transferability, the results will be given to people who have similar characteristics with the participants in order to compare the research findings and their experiences. In order to approve the confirmability of the data, the opinions of qualitative researchers will be sought outside the settings of the present research and the stages of the study will be recorded accurately, and the continuity of the work will be controlled regularly.

### Phase II: literature review

In the second phase of the study, the researcher will start searching literature and texts that address perinatal palliative care for fetal anomaly diagnosis.

At this stage, all studies with quantitative, clinical guidelines, service package, Program, protocol, qualitative or mixed method which have been published from 2000 to 2020 in English and Persian will be reviewed. The search is performed using keywords and different combinations including “Perinatal Palliative Care”, “Perinatal Service Package”, “Fetal Anomaly Diagnosis”, “Experiences of Fetal Anomaly”, “service package” and “Perinatal Loss Service”. Several multiple databases are available for searching the related papers, including Scopus, MEDLINE, Ovid, ProQuest, PubMed, Science Direct, Web of Science, Embase, Cochrane Library, Magiran, SID, Embase, and CINAHL.

### Phase III: designing a preliminary perinatal palliative care service package

In this phase, using the results of the qualitative study and reviewing the literature, a list of recommendations will be developed and prioritied. Then, a preliminary service package will be designed based on these data and sent to all of the experts who desire to participate.

### Phase IV

In this phase, the opinion of experts about this service package will be collected by using RAND/UCLA Appropriateness Method technique. Panel members include psychologists, Palliative specialist, obstetricians, geneticists, neonatologist, midwives, nurses, sonographists, perinatologist, reproductive health professionals, and maternal health policy makers.

The first round of each recomendation will be rated on a scale of 1–9 by experts. The mean score of 7 to 9 will be labeled as “appropriate”, the mean score of 1–3, “inappropriate,” the mean score of 4–6, “undetermined” by the experts. Also, the experts are allowed to add recommendations to this list based on their own knowledge and experience [29].

In the second round, after coordination and sending invitations to the experts,

the panel members will come together with the research team in a meeting and will hold group discussion [30].

The experts discuss the scoring of each recommendation, especially on the uncertain scores. The focus will be on the conflicting cases and there is an opportunity to revise of services and final decision will be made. In the end, considering the conclusions of the experts’ opinions and performing necessary revision, the final version of the perinatal palliative care service package for fetal anomaly diagnosis will be developed.

Then health providers will apply these service packages for 3 months. The applicability of the service package recommendations in clinical settings will be assessed.

### The study population and sampling method

Eighty-eight of the volunteer health providers include nurses, midwives, obstetricians, neonatologist and perinatologist in the hospitals, clinics and health centers with at least 5 years of experience in providing care to women with fetal or neonate anomaly diagnosis .

### Data collection method

Checklist is designed from the service packages recommendations with score of applicability of recommendation of 0 to 100. Recommendations that achieve a score above 70% are unchanged in the service packages, and recommendations that score below 70% are reviewed by experts.

Then, by concluding the health provider’s options, final version of perinatal palliative care service package will be provided.

## Discussion

This study is Developing of the First Service Package for women with Fetal Anomaly Diagnosis in Iran. The detection of fetal anomaly in pregnancy, particularly after passing fetal danger zone of the first trimester, is usually unexpected [31]. According to the results of the studies, pregnant women who are confronted with diagnosis of fetal anomalies report showed negative emotional responses such as grief during the time of the diagnosis, stress, anxiety, guilt and social isolation. When the pregnancy is continued, women express a sense of hope and worry, hope for normality and concern about the future and needs of the newborn [10].

Families in this situation need to obtain the desired information about pregnancy, delivery, and medical interventions. Thus, Perinatal Palliative Care program can be considered by the pregnancy counselors, and there should be more focus on the needs of women and their family at all aspects in the perinatal period, including: the time of diagnosis and decision making in pregnancy, throughout delivery planning, during the birth process, during the newborn’s life and death, and postmortem periods [15, 32].

Perinatal palliative care refers to a comprehensive, integrative coordinated care that comprises options for obstetric and newborn care that should be available to all families confronted with conditions considered to be life-limiting in fetal or early infancy [21, 33].

Additionally, this program can link together medical, psychological, spiritual and social support for the affected families [32, 34].

In order to develop any guideline, the needs, values, beliefs, as well as the ethical, spiritual and cultural context of the society should be considered [35].

Considering the lack of a comprehensive perinatal palliative care service package for these women in Iran, this study will be an attempt to find the perinatal palliative care needs and develop a service package. The results of this mixed methods study are expected to develop a service package with multi-professional and interdisciplinary approach that meets the needs of the women with perinatal anomaly diagnosis and in accordance with the socio-cultural context of the research population.

## Data Availability

Not applicable.

## Abbreviations

NICU: Neonatal intensive care unit
PPC: Perinatal palliative care
RAND: Research and Development
UCLA: University of California at Los Angeles
UROCAT: European Surveillance of Congenital Anomalies
